# Effects of *GS3* Editing in *japonica* Rice ‘Nipponbare’ on Grain Morphology, Yield Components, and Response to Heat Stress at the Reproductive Stage

**DOI:** 10.3390/plants14182897

**Published:** 2025-09-18

**Authors:** Yongbin Qi, Numphet Sangarwut, Keasinee Tongmark, Sriprapai Chakhonkaen, Linyou Wang, Amorntip Muangprom

**Affiliations:** 1 Institute of Crop Science and Nuclear Technology Utilization, Zhejiang Academy of Agricultural Sciences, Hangzhou 310021, China; qi_yongbin@hotmail.com (Y.Q.); ly217wang@sina.com (L.W.); 2 National Center for Genetic Engineering and Biotechnology, Thailand Science Park, Khlong Luang, Pathum Thani 12120, Thailand; numphet.san@biotec.or.th (N.S.); keasinee.ton@biotec.or.th (K.T.); meaw.meow14@gmail.com (S.C.)

**Keywords:** CRISPR/Cas9, *GS3*, *japonica*, grain size, heat tolerant, reproductive stage, chitinase-related genes

## Abstract

Rice (*Oryza sativa*), particularly the *japonica* subspecies, is a vital global food source but often suffers from short grain length and heat sensitivity, highlighting the need for genetic improvement. This study employed CRISPR/Cas9 technology to investigate the effects of *Grain Size3* (*GS3*) gene editing in the *japonica* cultivar, ‘Nipponbare’. Successful *GS3* editing increased grain size across stable T_3_ and T_4_ generations. Importantly, different *GS3*-edited lines, even when all targeted within exon 1, resulted in varied effects on grain length and other yield components. Transcriptomic analyses revealed unique gene expression profiles for each edited line, highlighting the fact that subtle *GS3* mutations trigger diverse transcriptional cascades. While common differentially expressed genes (DEGs) were enriched in ethylene signaling and chitinase activity, line-specific KEGG analyses showed distinct pathway enrichments. Crucially, the CR-L5 line exhibited significantly enhanced heat tolerance at heading stage. Under high-temperature stress, CR-L5 maintained a higher relative seed setting rate and a 15% greater grain yield than the wild type. This enhanced thermotolerance in CR-L5 correlated with differing expressions of several wax biosynthesis and chitinase-related genes. Our study provides evidence that specific *gs3* mutations can confer enhanced reproductive-stage thermotolerance, offering a strategy for breeding climate-resilient *japonica* rice with improved grain quality and yield under stress.

## 1. Introduction

Rice (*Oryza sativa*) is an important staple food globally, with over 90% of its cultivation and consumption concentrated in Asia [1,2]. Asian cultivated rice is classified into two geographically and genetically divergent subspecies: *O. sativa* ssp. *japonica* and *O. sativa* ssp. *indica*. The *japonica* subspecies is primarily cultivated in temperate regions at higher latitudes, such as northeastern China (Heilongjiang, Jilin, and Liaoning provinces), Japan, South Korea, California in the United States, and parts of southern Europe including Italy. In contrast, the indica subspecies is predominantly grown in subtropical and tropical regions at lower latitudes or altitudes, including South and Southeast Asia, particularly India, Bangladesh, Indonesia, and Thailand. This ecological separation has contributed to their distinct morphological, physiological, and genetic characteristics [3,4]. Among its diverse forms, the *japonica* subspecies holds particular significance. *Japonica* rice can be divided into two subgroups: temperate *japonica* and tropical *japonica*. Temperate *japonica* is more widely produced and consumed than tropical *japonica*. Many temperate *japonica* rice varieties are valued for their sticky texture and high grain quality, which are distinct from those of *indica* varieties. However, temperate *japonica* rice plants exhibit poor adaptability to tropical environments [5]. In general, *japonica* rice is highly sensitive to temperature variations. High temperatures, particularly during the reproductive stages (flowering and grain filling), can severely reduce yield and grain quality [6]. Due to this limitation, the production of temperate *japonica* rice has been very limited in tropical areas. Thus, it is crucial to develop new *japonica* cultivars that possess both high grain quality and resistance to high temperatures.

Nipponbare, a representative *japonica* cultivar, is critically important for rice genomics research, given its high-quality sequenced genome [7,8,9]. Furthermore, breeding efforts involving Nipponbare have successfully improved key agronomic traits like grain yield and quality [10,11]. However, a significant limitation of Nipponbare and many other temperate *japonica* varieties is their short grain length and susceptibility to high temperatures. This presents a challenge, as consumer preference strongly leans towards slender grain shapes, which are associated with higher-quality appearance [12,13]. Indeed, visual characteristics such as grain size, shape, and the presence of chalkiness profoundly influence consumer purchasing decisions [12].

Grain size is a primary determinant of grain weight, which directly impacts overall grain yield, alongside the number of panicles per plant and grains per panicle [14]. Decades of genetic research have illuminated the molecular basis of grain shape, identifying and cloning numerous genes and quantitative trait loci (QTL) that govern this trait [15]. Key genes controlling grain length include *GS3*, *qGL3*, *GL7/GW7*, *GL4*, *TGW3/GL3.3*, *GS9*, *GL10*, *GSW3.1* and *GSW3.2* [16,17,18,19,20,21,22,23,24,25]. Among these, *GS3* (*Grain Size3*) is particularly significant due to its consistent role in enhancing grain length when mutated, making it an attractive target for yield improvement [26].

The *GS3* locus on chromosome 3 has been consistently identified as a major quantitative trait locus (QTL) governing both grain weight and length, acting as a negative regulator of grain and organ size [16,26]. A pivotal single nucleotide polymorphism (SNP) in the second exon of *GS3* distinguishes long-grain varieties (TGA, a termination codon) from small-grain varieties (TGC, a cysteine codon) [27]. Extensive research confirms that the *gs3* allele and this C/A SNP are functional mutations leading to longer grains [27,28]. Recent advancements in CRISPR/Cas9-mediated gene editing have allowed for precise manipulation of *GS3*. For example, editing targets in the first and fifth exons of *GS3* increased panicle length and grain size [29]. While simultaneous editing of *GS3* and *GL3.1* in Nipponbare led to longer grains, it also reduced grain number and overall yield [30]. Conversely, knocking out *GS3* in the first exon enhanced both grain length and yield per plant [31]. These findings collectively demonstrate that manipulating *GS3* can consistently increase grain size, a desirable trait for yield. However, the exact impact of *gs3* knockout on other yield components can vary significantly, depending on the rice cultivar’s genetic background [13].

Beyond grain traits, climate change, particularly rising global temperatures, poses a severe and growing threat to rice yield and quality worldwide [32]. Therefore, identifying genetic resources that confer both improved yield and enhanced heat tolerance in a model *japonica* variety like Nipponbare is highly relevant and practical. Such discoveries could provide crucial solutions for adapting *japonica* rice cultivation to a warming climate. Intriguingly, recent studies have investigated the molecular mechanisms of thermotolerance involving natural variations in the G-protein γ subunits, GS3/TT2, in African and Asian rice. These studies reported that under heat stress, GS3 promotes an increase in cytosolic Ca^2+^ levels, fostering downstream interactions between calmodulin and the transcription factor SCT1. This interaction suppresses *OsWR2* gene expression, leading to reduced cuticular wax content and increased thermosensitivity. Conversely, loss-of-function *gs3* alleles disrupt this process, preventing Ca^2+^ accumulation and CaM–SCT1 interaction, thereby inducing *OsWR2* gene expression and promoting cuticular wax synthesis [33,34]. These findings suggest that *gs3* could be a valuable target for improving rice yield under high-temperature conditions. Indeed, [13] recently demonstrated that knocking out the *GS3* gene improved the thermotolerance of Geng/Japonica (GJ) rice varieties at the seedling stage under a hydroponic culture solution. While [13] recently reported improved seedling thermotolerance in broader Geng/Japonica varieties, the precise, integrated impact of *GS3* knockout on both desirable grain morphology and critical reproductive-stage yield components under heat stress in a genetically defined model *japonica* cultivar like Nipponbare remains a significant knowledge gap. Understanding this interplay is vital for climate-resilient rice breeding.

Against this background, our study provides critical advancement by investigating the pleiotropic effects of *gs3* mutation using CRISPR/Cas9 technology in the highly characterized *japonica* model cultivar, Nipponbare. Building on preliminary observations of increased grain length in mutant transgenic lines, our research aimed to quantitatively assess the comprehensive effects of *gs3* mutation on grain length and vital yield components across stable T_3_ and T_4_ generations and elucidate the underlying molecular mechanisms by conducting in-depth transcriptomic analyses to identify affected genes and pathways. Finally, we determined the direct impact of high-temperature treatment on the yield and yield components of *gs3* mutants in Nipponbare at agriculturally relevant growth stages, a critical area not fully addressed by previous work.

## 2. Results

### 2.1. Effects of GS3 Editing on Grain Length and Yield Components

To investigate the effect of *GS3* editing on yield components, a CRISPR/Cas9 construct targeting *GS3* was introduced into ‘Nipponbare’ (NB). Grain lengths of T_0_ transgenic plants were measured, and those exhibiting longer grains than NB were self-pollinated to obtain seeds. The resulting seeds were planted, and transgenic plants were selected and advanced by self-pollination up to the T_3_ generation.

**Figure 1:**
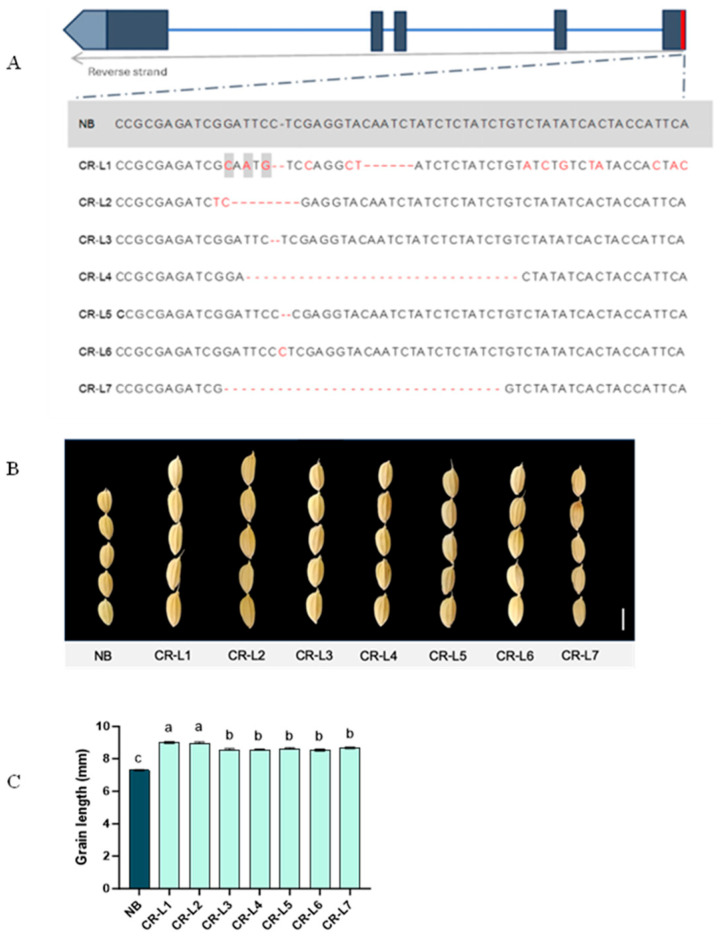
*GS3* sequences and effects of *GS3* on grain length. (**A**) Sequence comparison between wild-type Nipponbare (NB) and seven T_3_ *GS3*-edited plants that exhibit longer grain lengths. The wild-type sequence is highlighted in gray, while CR-L1 to CR-L7 represent the seven T_3_ *GS3*-edited lines. Red fonts represent the mismatch bases; hyphens represent deletion. (**B**) Representative images of grain shape from wild type and their *GS3*-edited plants. The scale bar represents 5 mm. (**C**) Grain length measurements for wild type and *GS3*-edited plants. Data are presented as mean ± standard error (SE), from 14–17 plants for edited lines and 10 plants for the NB control line. Different letters above the bars indicate significant differences (*p* < 0.05), determined by a Tukey’s multiple range test.

Seven T_3_ *GS3*-edited plants from different families were subjected to sequencing. All selected transgenic plants displayed distinct mutations within the *GS3* gene (Figure 1A). Regarding agronomic traits, all transgenic lines showed a significant increase in grain length, ranging from 8.37 mm to 9.18 mm, compared to NB’s grain length of 7.30 mm (Figure 1B,C).

**Figure 2:**
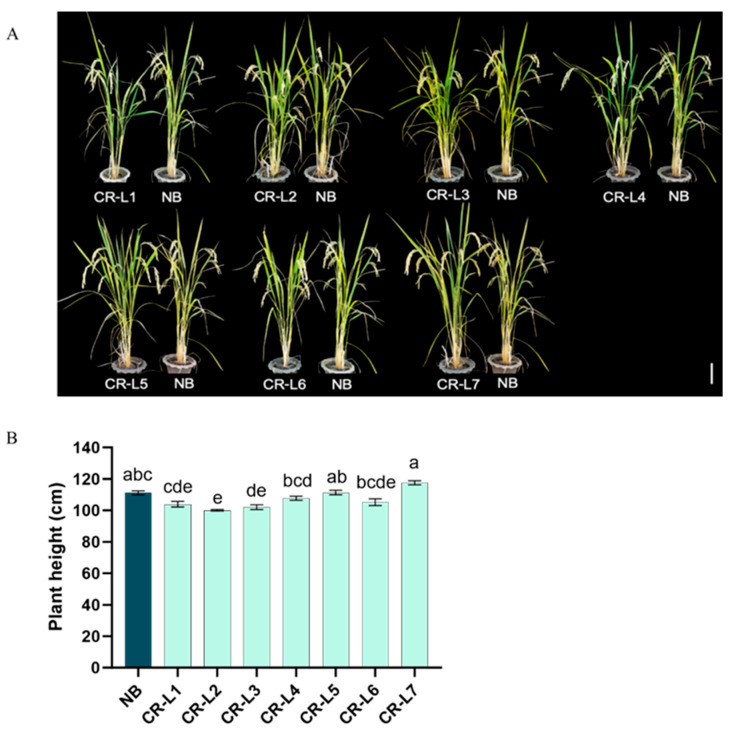
Effects of GS3 Editing on Plant Height. (**A**) Representative images of wild-type Nipponbare (NB) and *GS3*-edited plants, Scale bar = 20 cm. (**B**). Plant height of wild-type Nipponbare (NB) and *GS3*-edited plants. Data are presented as mean ± standard error (SE), from 14–17 plants. Different letters indicate significant differences (*p* < 0.05) based on a Tukey’s multiple range test.

Regarding the effects of *GS3* editing on plant height, *GS3* editing had a varied impact on plant height. While some of the edited plants showed significant differences, others were not significantly different from the non-edited control (NB). Specifically, CR-L1, CR-L2, and CR-L3 were significantly shorter than CR-L5 and CR-L7, though most of these lines showed no significant difference in height when compared to NB (Figure 2A,B).

**Figure 3:**
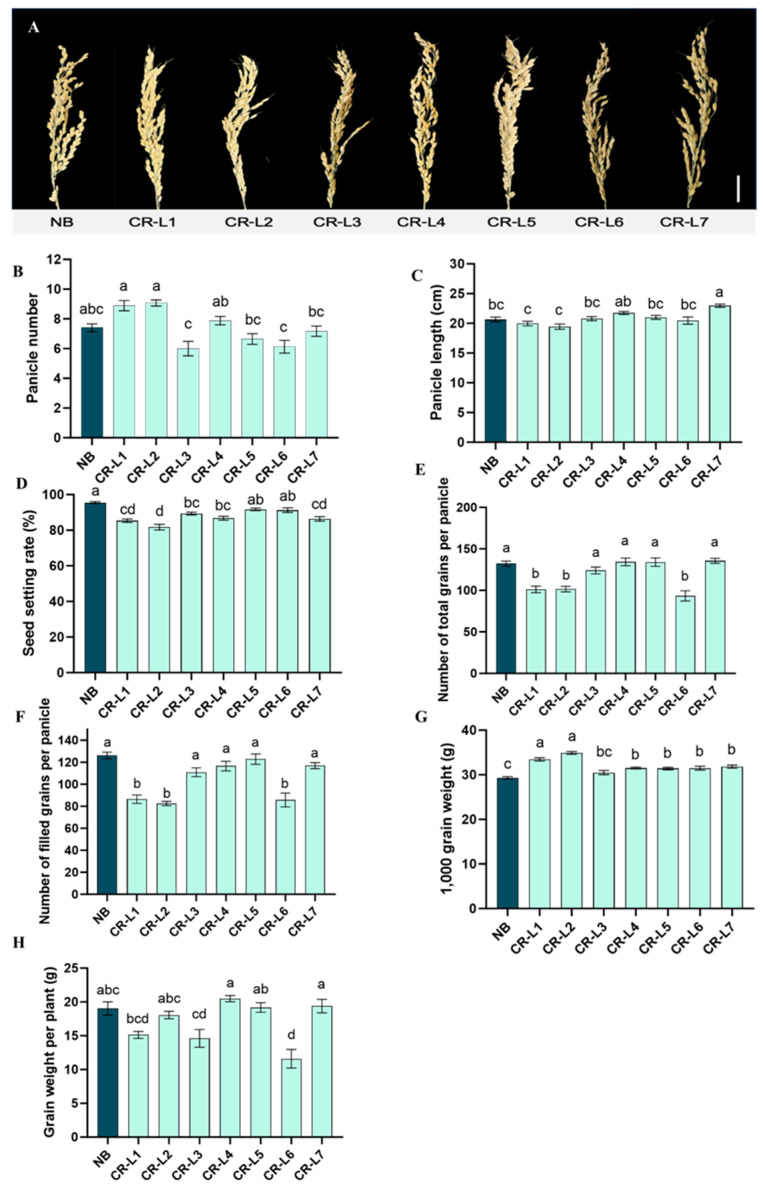
Effects of GS3 on yield-related traits. (**A**) Representative images of panicles of wild-type Nipponbare (NB) and GS3-edited lines. (**B**) Panicle number. (**C**) Panicle length. (**D**) Seed setting rate (%). (**E**) The number of filled grains per panicle. (**F**) The number of total grains per panicle. (**G**) 1000-grain weight. (**H**) Grain weight per plant. Scale bar = 20 cm. Data are presented as mean ± standard error (SE), from 14–17 plants for edited lines and 10 plants for NB control line. Different letters indicate significant differences (*p* < 0.05) based on a Tukey’s multiple range test.

Significant differences in yield components were found between the *GS3*-edited plants and the non-edited control (NB) (Figure 3A–H). Editing the *GS3* gene led to a significant increase in panicle length for line CR-L7, compared to NB (Figure 3A,C). This line’s panicle length was also longer than all other tested lines, except for CR-L4.

Regarding panicle number, lines CR-L1 and CR-L2 had significantly more panicles than CR-L3, CR-L5, CR-L6, and CR-L7, but none of the edited lines differed significantly from NB (Figure 3B).

Conversely, all tested *GS3*-edited lines showed no significant positive effect on seed setting rate, the number of filled grains per panicle, or the number of total grains per panicle (Figure 3D–F). In fact, lines CR-L1, CR-L2, and CR-L7 had significantly lower seed setting rates than CR-L5, CR-L6, and NB (Figure 3D). Additionally, CR-L1, CR-L2, and CR-L6 had fewer filled and total grains per panicle than most other lines and NB (Figure 3E,F).

Notably, the editing had a strong positive effect on 1000-grain weight, which was significantly increased in all edited lines except CR-L3, compared to NB (Figure 3G). Despite this, there was no significant positive effect on grain weight per plant between the edited lines and NB, although CR-L4 CR-L5 and CR-L7 did have higher grain weight per plant than NB (Figure 3H).

### 2.2. Identification of DEGs Between Wild-Type and GS3-Edited Lines and Prediction of Their Functions

**Figure 4:**
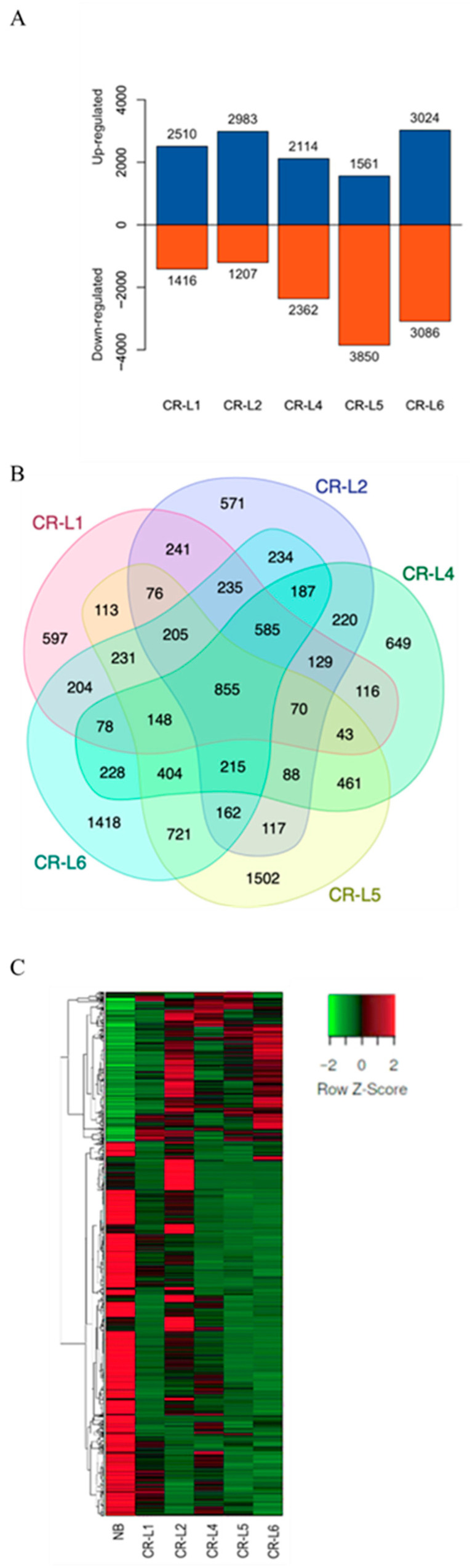
Transcriptome analysis. Differentially expressed genes (DEGs) in each GS3-edited line were identified by comparison with the wild-type Nipponbare (NB). (**A**) The number of upregulated and downregulated DEGs in each *GS3*-edited line. (**B**) Venn diagram presenting co-expression of DEGs expressed in each sample, with overlapping regions indicating the number of genes expressed in all samples. (**C**) Hierarchical clustering of DEGs expressed in wild type (NB) and their *GS3*-edited lines, based on FPKM values. Blue and red represent upregulated and downregulated DEGs, respectively, and the numbers on the columns indicate the count of DEGs. Green represents lower expression, while red represents higher expression. Columns indicate individual experiments, and rows indicate DEGs.

To further examine expression changes between wild type and *GS3*-edited lines with potential phenotypic improvements, five *GS3*-edited lines, including CR-L1, CR-L2, CR-L4, CR-L5, and CR-L6, were selected. Differentially expressed genes (DEGs) were identified in comparison with Nipponbare (NB). The results showed 3926 DEGs in CR-L1, 4190 in CR-L2, 4476 in CR-L4, 5411 in CR-L5, and 6110 in CR-L6, respectively (Figure 4A).

A total of 855 DEGs were commonly expressed in all samples, while 597, 571, 649, 1502, and 1418 genes were specifically expressed in CR-L1, CR-L2, CR-L4, CR-L5, and CR-L6, respectively (Figure 4B). These results indicated that variability in gene expression exists between NB and each of the *GS3*-edited lines (Figure 4C).

To predict the functions of both the 855 common DEGs and the specific DEGs in each *GS3*-edited line, Gene Ontology (GO) and Kyoto Encyclopedia of Genes and Genomes (KEGG) analyses were performed. The top ten significant GO terms and KEGG enrichments were collected based on a *p*-value (*p*-adj) ≤ 0.05 and a false discovery rate (FDR) ≤ 0.05.

GO enrichment analysis indicated that the 855 common DEGs were significantly enriched in biological processes (BPs) related to the ethylene hormone, including the ethylene-activated signaling pathway, cellular response to ethylene stimulus, and response to ethylene (Figure S2A). For cellular component (CC), the most enriched term was apoplast, which is associated with programmed cell death (Figure S2B). In terms of molecular function (MF), the common DEGs showed enrichment in chitinase activity (Figure S2C).

For the GO analysis of specific DEGs within each CR-L line, distinct enrichments were observed in BP. Both CR-L1 and CR-L2 showed similar enrichments for “response to water deprivation” and “water response,” suggesting a role in water stress pathways. In contrast, “hydrogen peroxide catabolic process” and “regulation of transcription DNA-templated” were enriched in both CR-L4 and CR-L5 (Figure S3A–D), indicating involvement in oxidative stress and gene regulation. The “regulation of macromolecule biosynthetic process” was uniquely identified in the CR-L6 line (Figure S3E). Regarding CC, “apoplast” and “extracellular region” were enriched in CR-L1, CR-L2, and CR-L4. However, in CR-L5, “anchored components of membrane” were enriched, while “plant-type cell wall” components were enriched in CR-L6 (Figure S4). For MF, chitinase activity was the most highly enriched term across all CR-L lines (Figure S5), which suggests a broad involvement of chitinases in the responses triggered by GS3 editing.

KEGG pathway analyses revealed that 11 of the 855 common DEGs were significantly enriched in pathways related to amino sugar and nucleotide sugar metabolism (Table 1). Among these eleven genes, six showed particularly high expression in the CR-L5 line: *Os01g0660200* (Acidic class III chitinase OsChib3a precursor), *Os05g0247100* (similar to Glycosyl hydrolases family 18), *Os11g0462100* (Glycoside hydrolase, family 18 protein), *Os11g0700900* (Glycoside hydrolase, subgroup, catalytic core domain-containing protein), *Os11g0701400* (Chitinase (EC 3.2.1.14) III C10150-rice), and *Os11g0702100* (similar to Class III chitinase homologue). Additionally, *Os07g0446800* (similar to Hexokinase) and *Os11g0462100* (Glycoside hydrolase, family 18 protein) exhibited high expression across all CR-L lines when compared to Nipponbare (NB) (Table 1).

**Figure 5:**
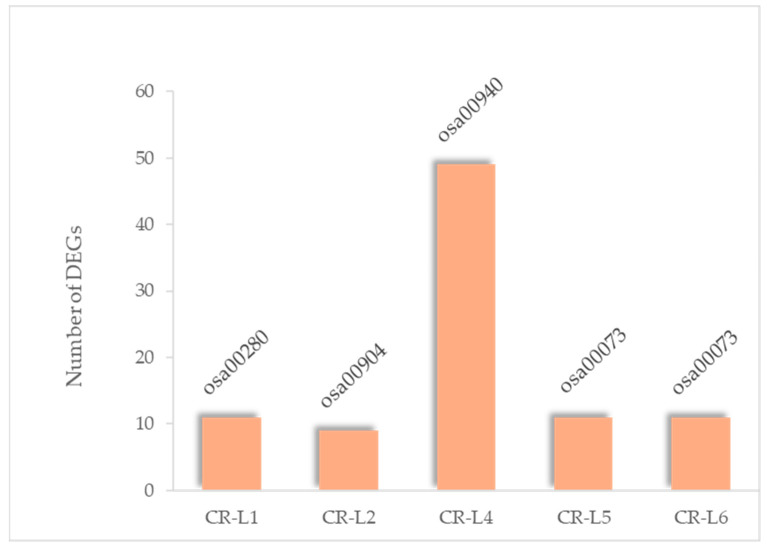
Highest fold-enrichment KEGG pathways of DEGs identified in each CR-L line.

The expression profiles of genes assigned to the highest fold-enrichment pathways were further analyzed for each *GS3*-edited CR-L line. The highest fold enrichment of each CR-L line was 11 genes assigned to osa00280 (valine, leucine, and isoleucine degradation) in the CR-L1 line; 9 genes assigned to osa00904 (diterpenoid biosynthesis) in the CR-L2 line; 49 genes assigned to osa00940 (phenylpropanoid biosynthesis) in the CR-L4 line; and 11 genes each assigned to osa00073 (cutin, suberin, and wax biosynthesis) in both the CR-L5 and CR-L6 lines (Figure 5; Supplementary Table S1). All of the genes in CR-L1 were upregulated. A similar trend was observed in CR-L2, except for *Os06g011000*, which was downregulated. Several of these genes exhibited strong upregulation. Interestingly, in CR-L4, most of the genes belonged to the class III peroxidase family and displayed both strong up- and downregulation. For CR-L5 and CR-L6, the DEGs were enriched in the same pathway; however, there were notable differences. *Os04g0511200* (EF-hand, abscisic acid–responsive 27-kDa protein) was highly upregulated in CR-L6, but downregulated in CR-L5. Additionally, *Os06g0254600* was included in the most highly enriched pathway in CR-L5 but not in CR-L6, whereas *Os03g0167600* was present in the most highly enriched pathway in CR-L6 but not in CR-L5. Notably, most of the genes in CR-L5 and CR-L6 were downregulated (Supplementary Table S1).

### 2.3. Effects of GS3 on Yield and Yield Components Under High-Temperature Treatment

Wild-type Nipponbare and four selected T_4_ *GS3*-edited lines including CR-L2, CR-L5, CR-L6 and CR-L7 were used to assess the effects of high temperature (38/30 °C for 7 days compared to normal condition, 28/22 °C) on yield and yield components using artificial climatic chambers. The control and treatment groups were grown under natural conditions. The high-temperature treatment was applied for 7 days, beginning at the heading stage.

Under normal conditions, the number of panicles per pot, number of filled grains per plant, total grain number per plant, and seed setting rate in all *GS3*-edited lines were not higher than those in the wild-type Nipponbare. However, the *GS3*-edited lines showed increased grain number per panicle and 1000-grain weight, though not all differences were statistically significant. In terms of grain yield per pot, CR-L2 and CR-L5 outperformed the wild type, while CR-L6 and CR-L7 yielded less (Table 2).

Under high-temperature stress, panicle number per pot, grain number per panicle, and total grain number per plant were not significantly affected in any tested line. However, filled grain number per plant, seed setting rate, and grain yield per pot declined across all tested lines. Notably, CR-L5 exhibited a higher seed setting rate than the wild type under stress, although this difference was not statistically significant. Notably, the relative seed setting rate of CR-L5 (58.81%) was significantly higher than that of the wild type and the other edited lines. Moreover, CR-L5 showed approximately 15% higher grain yield than the wild type, whereas the other *GS3*-edited lines yielded less. Interestingly, under high-temperature stress, all *GS3*-edited lines had significantly higher grain number per panicle than the wild type. Additionally, CR-L7 demonstrated a significantly greater 1000-grain weight than the wild type (Table 2).

## 3. Discussion

### 3.1. The Pleiotropic and Allele-Specific Effects of GS3 Mutations on Agronomic Traits

Consumer preference for elongated, slender rice grains significantly influences market value and purchasing decisions [12]. Our study confirms the well-established role of the *GS3* gene as a potent negative regulator of grain size [16,26,31]. All seven independent CRISPR/Cas9-edited T_3_ lines, each carrying a distinct mutation in *GS3*, exhibited a significant increase in grain length and 1000-grain weight compared to the wild-type Nipponbare (NB). This finding aligns perfectly with established knowledge and highlights the effectiveness of *GS3* as a target for breeding programs aimed at improving grain appearance, a trait that significantly influences consumer preference and market value [12,13,31].

However, a key takeaway from our research is the extensive phenotypic diversity observed among these edited lines, even though they all have mutations within the same exon. The results showed that the main effects of *GS3* editing on grain length and grain weight were highly significant. Notably, *GS3* editing may exert minor effects on other phenotypic variations. This reveals that a specific mutation can have significant pleiotropic effects on other important agronomic traits. For example, while all lines had longer grains, their plant height varied: CR-L1, CR-L2, and CR-L3 were significantly shorter than CR-L5 and CR-L7. This demonstrates that different mutations can differentially impact plant architecture. While off-target effects could not be completely ruled out, off-target effects were minimized by designing gRNAs with unique target sequences and using a high-fidelity Cas9 variant. Potential off-target sites were further assessed by in silico prediction. Therefore, the observed differences are most likely attributable to *GS3* editing. Indeed, the results showed that each line with distinct sequence mutations exhibited corresponding differences in phenotypes and gene expression.

It should be noted that in this study, sequencing validation was performed only at the *GS3* target region. Although additional sequencing of predicted off-target sites or whole-genome resequencing would provide further confirmation, such analyses were beyond the scope of the present work. Importantly, some published CRISPR/Cas9 studies in rice have adopted a similar approach of validating only the target-site mutations [35,36,37]. Future research incorporating more extensive off-target assessment, including whole-genome sequencing, will be valuable to further strengthen confidence in the precision of *GS3* editing.

This allele-specific effect was even more evident in yield components. Panicle length increased significantly only in line CR-L7, while panicle number increased in CR-L1 and CR-L2. This suggests that the specific mutations in these lines may uniquely affect panicle development, a trait not consistently altered across all edited lines. This variability, depending on the specific *gs3* allele and genetic background, has been previously noted [13].

Crucially, the increase in grain size did not consistently translate to higher overall yield under normal conditions. In fact, we observed a potential trade-off, as seed setting rate, the number of filled grains per panicle, and the number of total grains per panicle were not positively affected across all lines, and were even significantly lower in lines like CR-L1 and CR-L2 compared to the wild type and other edited lines. Despite the universal increase in 1000-grain weight in most lines, grain weight per plant was not significantly higher than NB, except for CR-L4, CR-L5 and CR-L7. This underscores the need to characterize specific mutant alleles to avoid negative pleiotropic effects on yield, a finding consistent with prior work by [30].

### 3.2. Molecular Basis of Phenotypic Variation: Insights from Gene Expression

To unravel the molecular mechanisms underlying these diverse phenotypes, we performed transcriptome analysis on five selected edited lines (CR-L1, CR-L2, CR-L4, CR-L5, and CR-L6). CR-L7 was excluded because it carried sequence mutations very similar to those of CR-L4, and thus was not expected to provide additional transcriptomic insights. The results were that each line showed a distinct set of differentially expressed genes (DEGs), with the number of DEGs ranging from 3926 in CR-L1 to 6110 in CR-L6. This variability in gene-expression profiles directly correlates with the phenotypic differences observed among the lines, and demonstrates that even subtle mutations within *GS3* can trigger unique downstream transcriptional cascades.

GO analysis provided further insights into these distinct genetic responses. While a core set of 855 common DEGs showed enrichment in pathways related to ethylene signaling and chitinase activity across all lines, the line-specific DEGs revealed unique functional enrichments. For example, CR-L1 and CR-L2 showed enrichment in stress pathways related to water deprivation. In contrast, CR-L4 and CR-L5 showed enrichment in processes related to oxidative stress and transcription regulation. These findings suggest that the specific mutation in each line triggers a unique compensatory response in gene expression, leading to their distinct phenotypes.

The KEGG analysis showed that 11 out of the 855 common DEGs were significantly enriched in pathways related to amino-sugar and nucleotide-sugar metabolism, including genes associated with chitinase and its precursor. However, the KEGG analysis of DEGs specific for each line further highlighted the specificity, with each line showing the highest enrichment in different metabolic pathways. For example, CR-L1 showed enrichment in amino acid degradation, CR-L2 in diterpenoid biosynthesis, and CR-L4 in phenylpropanoid biosynthesis. Most notably, both CR-L5 and CR-L6 showed a significant enrichment in the cutin, suberin, and wax biosynthesis pathway, which is associated with heat tolerance. This finding is particularly interesting, given recent reports that link *GS3* mutations to enhanced thermotolerance through increased cuticular wax content [13,33,34]. Despite this shared enrichment, differences in gene numbers and expression levels within this pathway between CR-L5 and CR-L6 may explain their varying heat tolerance. Furthermore, among the eleven common DEGs, six exhibited the highest expression in CR-L5, with three of these being chitinase-related genes. The elevated expression of chitinase-related genes in CR-L5 suggests a potential link to its observed phenotypes. Accordingly, although the primary function of chitinase in rice is recognized for biotic stress defense, there are indications and broader plant studies suggesting its involvement in abiotic stress responses, including heat [38,39].

While our transcriptome analysis provided valuable insights into allele-specific expression patterns and enriched pathways, we acknowledge that additional statistical validation—such as Pearson correlation and principal component analyses—would further strengthen the assessment of sample reliability and clustering quality. Due to current constraints, these analyses were not performed in the present study. Nevertheless, the clear and biologically meaningful differences observed among the edited lines, together with the consistency between transcriptomic profiles and phenotypic outcomes, support the robustness of our findings. Future studies incorporating correlation and clustering analyses will be valuable to confirm and extend these interpretations.

### 3.3. Allele-Specific Thermotolerance at the Reproductive Stage

Under normal conditions, our results from T_3_ and T_4_ generations confirm the expected function of *GS3* editing in increasing grain size [13,31]. The most significant and novel finding of our research, however, lies in the differential response of these edited lines to high-temperature stress during the crucial heading stage—an agriculturally vital and heat-sensitive developmental period [6]. While a general decline in yield components was observed across all lines under heat stress, line CR-L5 exhibited a significantly higher relative seed setting rate and a 15% higher grain yield per pot than the wild-type Nipponbare. This superior performance was not observed in the other edited lines (CR-L2, CR-L6, and CR-L7), which all yielded less than the wild type. This result provides strong genetic evidence that the specific *gs3* mutation in CR-L5 confers enhanced thermotolerance at the reproductive stage. This is a crucial distinction from previous studies that focused on seedling thermotolerance [13]. This result perfectly aligns with our gene expression analysis, which showed CR-L5 and CR-L6 were specifically enriched for pathways involved in wax biosynthesis, a known contributor to heat stress tolerance [33]. While both lines showed this enrichment, CR-L5’s superior phenotype suggests its unique mutation and subsequent gene expression profile may be more effective in enhancing thermotolerance. Specifically, the differing expressions of several wax-biosynthesis and chitinase-related genes in CR-L5 may directly contribute to its superior performance, highlighting a powerful link between a specific gene mutation, its transcriptional consequences, and a desirable agronomic trait under stress.

## 4. Materials and Methods

### 4.1. Plant Materials

Wild-type *Oryza sativa* L. ssp. *japonica* ‘Nipponbare’ served as the genetic background for *GS3* gene editing. Transgenic plants were cultivated in pots within a greenhouse at the National Science and Technology Development Agency, Pathum Thani Province, Thailand, up to the T_3_ generation. In each generation, 20 plants per family were grown and self-pollinated. Transgenic lines exhibiting longer grains than ‘Nipponbare’ were selected for advancement. From the T_3_ generation, seven *GS3*-edited lines with elongated grains were chosen for assessing yield and yield components under greenhouse conditions, as well as for transcriptome analysis. T_4_ plants derived from these seven selected T_3_ lines were subsequently subjected to high-temperature treatment in artificial climatic chambers.

### 4.2. Construction of Vectors and Plant Transformation

To produce the *GS3* gene editing, one pair of specific target primers near the 5′ of *GS3* gene location were selected to generate CRSIPR/Cas9 vectors. The specific primers were designed using the web-based tool (https://crispr.cos.uni-heidelberg.de/index.html, accessed on 5 July 2019). The PAM sequence was selected within the first exon of the *GS3* gene. Two target adaptors were first ligated into the pYLgRNA-OsU6a vector, respectively, and then connected into the pYLCRISPR/Cas9-MH vector. The detailed method of vector construction was described previously [40]. The final construct vector was transformed into *Agrobacterium tumefaciens* strain EHA105 by electroporation. Transgenic plants were generated by the rice transformation, using the method described preciously [41]. The *GS3* gene-edited lines were analyzed by the Sanger sequencing (BGI, Shenzhen, China) using the specific primers of GS3C-F1/R1. The primers were created using the sequences located at 400 base pairs before and after the specific target site (Table S1).

### 4.3. Assessment of Yields and Yield Components of T3 GS3-Edited Lines

To assess yield and yield components, wild-type ‘Nipponbare’ and T_3_ *GS3*-edited lines were cultivated in an air-conditioned greenhouse (28 ± 5 °C) with supplemental lighting on cloudy days. These plants were grown individually in pots at the National Center for Genetic Engineering and Biotechnology, part of the National Science and Technology Development Agency in Pathum Thani Province, Thailand. Planting started on 21 December 2021. For each line, 20 plants were grown in individual pots. From each T_3_ line, 14–17 plants were used to determine yields and yield components. This assessment took place 45 days after heading, following the protocols established by [42].

### 4.4. Transcriptome Analysis

A transcriptome assay was performed on wild-type ‘Nipponbare’ and their T_3_ *GS3*-edited lines to identify downstream genes of *GS3* involved in regulating grain size, grain quality, and thermotolerance. Total RNA was extracted from young panicles (2–4 cm) at the booting stage of both wild type and *GS3*-edited lines. For each line, three biological replicates were prepared from nine young panicles (three panicles per plant), using the RNeasy Plant Mini Kit (QIAGEN, Darmstadt, Germany), and subsequently pooled. The resulting libraries were sequenced using the Illumina HiSeq™ 2000 platform at Novogene Co., Ltd. (Beijing, China).

Raw sequence data have been deposited in the NCBI database under accession number PRJNA1308765. High-quality paired-end reads were mapped to the Nipponbare reference genome (IRGSP1.0) using HISAT2 [43]. Gene expression levels were calculated as Fragments Per Kilobase of transcript per Million mapped reads (FPKM) values. Differentially expressed genes (DEGs) between wild type and *GS3*-edited lines were analyzed using the EdgeR (version 4.4.1) R package. The analysis employed TMM (Trimmed Mean of M-values) normalization, a negative binomial distribution model, and a cut-off for DEGs set at a ∣log2(Fold change)∣ ≥ 1 and a *p*-value ≤ 0.05 [44]. DEGs were annotated for Gene Ontology (GO) terms, specifically in Biological Processes (BPs), Cellular Components (CCs), and Molecular Functions (MFs), with a corrected *p*-value ≤ 0.05 and a False Discovery Rate (FDR) ≤ 0.05. Kyoto Encyclopedia of Genes and Genomes (KEGG) pathway analysis was conducted using ShinyGO v0.75 [45].

### 4.5. High-Temperature Treatment Experiment

A high-temperature treatment experiment was conducted using artificial climatic chambers. Rice seeds were sown on 15 June 2024 and transplanted on 6 July 2024. Compound fertilizer was applied to the pots one day before transplanting the seedlings. Seedlings were transplanted into plastic buckets (two seedlings per bucket), with nine buckets (eighteen plants) used for each line. Each bucket contained 11 kg of dry bluish soil sourced from the local field. The soil composition was as follows: organic matter 36.5 g/kg, total nitrogen 2.75 g/kg, total phosphorus 0.63 g/kg, total potassium 20.3 g/kg, alkali-hydrolyzed nitrogen 236 mg/kg, ammonium nitrogen 9.6 mg/kg, available phosphorus 24.2 mg/kg, and available potassium 62 mg/kg. The soil pH was 6.5.

Before initiating the heat treatment, both control and treatment groups were grown under natural conditions. The high-temperature treatment started at the beginning of the heading stage of rice. Two temperature treatments were set: (1) control (CK), with an average temperature of 28/22 °C; and (2) high-temperature treatment, with an average temperature controlled at 38/30 °C for 7 days. After the seven-day heat treatment, both groups were returned to a normal environment until maturity. Before relocating the plants, panicles that had completed flowering were tagged, and only these tagged panicles were harvested upon maturity. The relative seed setting rate was then used to evaluate the heat resistance of the rice varieties.

### 4.6. Statistical Analysis

Statistical analysis was performed to compare traits between edited lines and the Nipponbare control line. Significant differences were assessed using one-way ANOVA (SPSS 17.0 software) followed by Tukey’s test for multiple comparisons. For clarity, statistically significant differences are indicated with different letter designations.

## 5. Conclusions

Our study demonstrates that CRISPR/Cas9-mediated editing of *GS3* consistently increases grain size, and the specific mutation generated can lead to variable and unpredictable pleiotropic effects on other agronomic traits. We identified a *gs3* allele in the CR-L5 line that confers superior reproductive-stage thermotolerance, resulting in significantly higher grain yield under heat stress. Transcriptomic analysis revealed differential expression of key genes, including those involved in wax biosynthesis and chitinase activity, which may underline this advantage. Although we did not perform additional sequencing to identify potential off-target mutations, our results provide genetic evidence that a specific *gs3* allele can enhance thermotolerance. These findings highlight the need for allele-specific characterization when using genome editing and provide a framework for developing high-yielding, heat-resilient *japonica* rice varieties through precise gene-editing strategies.

## Figures and Tables

**Table 1:** Eleven common differentially expressed genes (DEGs) in all CR-L lines in amino sugar and nucleotide sugar metabolism and their expression value in RNA seq.

Gene ID	Gene	Gene Annotation	RNAseq					
			NB	CR-L1	CR-L2	CR-L4	CR-L5	CR-L6
Os01g0860400	-	Similar to Acidic endochitinase precursor (EC 3.2.1.14)	1.96	0.66	0.67	0.89	0.93	0.59
Os01g0860500	*C10501*	Similar to Hevamine A precursor [Includes Chitinase (EC 3.2.1.14); Lysozyme (EC 3.2.1.17)]	8.26	1.28	0.07	0.27	0.87	0.18
* Os01g0660200	*C10728, OsChib3a*	Acidic class III chitinase OsChib3a precursor (Chitinase) (EC 3.2.1.14)	0.58	0.12	0.03	0.00	3.42	0.03
* Os05g0247100	Drought-induced protein 3, Xylanase inhibitor protein 2	Similar to Glycosyl hydrolases family 18	27.40	5.64	12.48	7.22	335.3	2.99
Os07g0446800	*HEXOKINASE-1*	Similar to Hexokinase	0.47	25.94	19.37	2.17	22.41	1.98
Os08g0518900	*C10122*	Chitinase (EC 3.2.1.14)	8.14	0.24	0.03	0.29	1.42	0.03
* Os11g0462100	-	Glycoside hydrolase, family 18 protein	0.90	2.08	2.79	9.68	12.72	6.87
* Os11g0700900	*C10923*	Glycoside hydrolase, subgroup, catalytic core domain-containing protein	1.05	0.23	0.26	0.00	18.06	0.06
* Os11g0701400	*C10150*	Chitinase (EC 3.2.1.14) III C10150-rice (EC 3.2.1.14)	0.82	0.00	0.00	0.00	4.01	0.00
Os11g0701800	xylanase inhibitor protein, rice xylanase inhibitor	Chitinase (EC 3.2.1.14) III C10701-rice (EC 3.2.1.14) (Class III chitinase homologue)	29.92	2.82	2.51	3.24	8.60	0.28
* Os11g0702100	-	Similar to Class III chitinase homologue	1.65	0.13	0.00	0.00	10.02	0.00

* Indicates genes showing the highest expression in CR-L5.

**Table 2:** Effects of high-temperature treatment on yield and yield components of *GS3*-edited lines.

Line	Treatment	Panicles Number/Pot	Grain Number /Panicle	Filled Grains Number/Plant	Total Grains Number/Plant	Seed Setting Rate (%)	Relative Seed Setting Rate (%)	1000-Grain Weight (g)	Yield (g/Pot)
*Nipponbare*	Normal	21 ± 2.65 a	74 ± 4.33 cd	1293 ± 263.04 a	1412 ± 108.89 ab	75.33 ± 2.42 ab	44.65 ± 5.27 b	20.97 ± 0.53 c	21.57 ± 1.80 bc
	High temp.	19 ± 0.58 a	64 ± 2.31 d	424 ± 35.87 c	1262 ± 23.23 ac	31.76 ± 0.16 d		23.27 ± 0.21 bc	9.84 ± 0.92 e
*CR-L2*	Normal	15 ± 2.65 ab	91 ± 7.48 ad	1063 ± 89.53 ab	1354 ± 123.92 a	78.60 ± 3.79 a	0.68 ± 0.55 c	24.60 ± 0.43 ab	26.15 ± 2.69 a
	High temp.	13 ± 2.65 b	94 ± 6.37 abc	2 ± 0.50 d	1406 ± 135.06 ab	0.51 ± 0.49 e		10.00 ± 0.60 e	0.03 ± 0.01 f
*CR-L5*	Normal	10 ± 1.00 b	118 ± 12.09 a	952 ± 67.18 ab	1183 ± 33.94 c	68.78 ± 3.17 bc	58.81 ± 13.94 a	24.33 ± 0.78 ac	23.47 ± 0.90 b
	High temp.	9 ± 2.08 b	113 ± 6.70 ab	467 ± 90.37 c	1228 ± 251.02 c	39.95 ± 0.93 d		24.36 ± 1.37 ac	11.32 ± 1.84 e
*CR-L6*	Normal	11 ± 2.12 b	96 ± 21.33 abc	709 ± 0.71 bc	1085 ± 41.01 c	65.44 ± 2.54 c	1.22 ± 1.13 c	22.68 ± 0.61 bc	16.10 ± 0.45 d
	High temp.	11 ± 0.00 b	98 ± 6.17 abc	8 ± 7.78 d	1080 ± 67.88 c	0.81 ± 0.77 e		15.60 ± 3.20 d	0.15 ± 0.15 f
*CR-L7*	Normal	13 ± 1.73 b	85 ± 12.94 bd	799 ± 42.39 b	1139 ± 79.20 bc	72.92 ± 2.42 ac	1.50 ± 2.07 c	23.49 ± 0.74 ac	18.76 ± 0.50 cd
	High temp.	12 ± 2.65 b	93 ± 10.43 abc	2 ± 3.54 d	1100 ± 137.49 ac	1.10 ± 1.54 e		27.29 ± 1.82 a	0.38 ± 0.54 f

Different letters in the same column indicate significant differences (*p* < 0.05) based on a Tukey’s multiple range test.

## Data Availability

Data will be made available on request. During the preparation of this work, the authors used Gemini (Gemini 2.5 Flash) and ChatGPT (GPT-4 and GPT-5 versions by OpenAI) in the writing process to improve the readability and language of the manuscript. After using this tool/service, the authors reviewed and edited the content as needed and take full responsibility for the content of the published article.

## Supplementary Materials

The following supporting information can be downloaded at: https://www.mdpi.com/article/10.3390/plants14182897/s1, Figure S1: Target sites and adjacent genomic sequences of GS3. Figure S2: The GO analysis of 855 common DEGs of all tested CR-L lines. Figure S3: The GO analysis of DEGs specific to each GS3-edited line assigned to the biological process. Figure S4: The GO analysis of DEGs specific to each GS3-edited line assigned to the cellular component. Figure S5: The GO analysis of DEGs specific to each GS3-edited line assigned to molecular function. Figure S6: The KEGG analysis of DEGs specific to each GS3-edited line assigned to molecular function. Table S1: The highest fold enrichment of DEGs in each CR-L line assigned to a pathway using Kyoto Encyclopedia of Genes and Genomes (KEGG) pathway database.
