# Supplementary material for: Effects of GS3 Editing in japonica Rice ‘Nipponbare’ on Grain Morphology, Yield Components, and Response to Heat Stress at the Reproductive Stage
Source: Plants (Basel). 2025 Sep 18;14(18):2897. doi: 10.3390/plants14182897 (PMC12473382; doi:10.3390/plants14182897)
Supplement: Supplementary file 1 [file plants-14-02897-s001.zip › plants-3830458-supplementary.pdf]

```
CTACTCGTTGGAAGTGTGCGTCTGCATGCATTATTGAACGGCTCTGATCCCCGCGGCGCAGCGGATCGGGGTCA
TGTCCGGATGGGCATATCGACGAGAAGGATCCGTCCCCGACAATCTTTCAAGGCCCGTGCCCCCGTCCCTCCTC
TCCTCTGCGCCTTTCCATCATCATTTACGCCCAACCCCAACACATGTACATTTCCCTTGGCTTGCTTCCGGAGA
AGAAAAGAGCGGCCATCCACTCCACTCTCCACTCTCTCCCTTCCATCATTACTTGCCCAAAAACGGCAATCCCC
TCCCCTCCATCTCCATGTGCTCTTCCACCTAGCTCCGCCATTCAAAGCAAAGCACCAAGCTTTTGCCTCTCCCT
CTACCATGCCTGCCCCCTATACATAGCTGCTGCACCGTCTCTCTTCATAAATATACTAGTAGGAGTAGCAGAGC
TCATCACTTCGATCATCTCCATTATCGGAACTTCGGAGTGACATGGCAATGGCGGCGGCGCCCCGGCCCAAGTC
GCCGCCGGCGCCGCCCGACCCATGCGGCCGCCACCGCCTCCAGCTCGCCGTCGACGCGCTCCACCGCGAGATCG
GATTCCTCGAGGTACAATCTATCTCTATCTGTCTATATCACTACCATTCATACTCCTTCGATCTTGCTTCAAAA
CAAAAAAATATATATTTCCTACTTCATATTCATATACACACGTACGGCTTGCTATCTGTCGAATTGTTTGCTTC
TGCATGCATGCATCACTCTCATTGTAAGTTTTTCCCAGCTTAAAACCACTCCTTTTATCTTCGTTCTTCTTCCT
TCTTGTTTTTTTTTAAAAAAACAACAACTCATTTAATCTTCATATAGTGTATCATGCATCATTTGCTTCTTTGA
TCAGTTCCCCAAAAACTGCTCCTCTCTTCCCAGCCAAA
```

Figure S1. Target sites and adjacent genomic sequences of *GS3*. The red region, the PAM (NGG) sequences. The yellow region, the target sites. The grey regions, the primer *GS3*C-F1 and *GS3*C-R1, respectively.

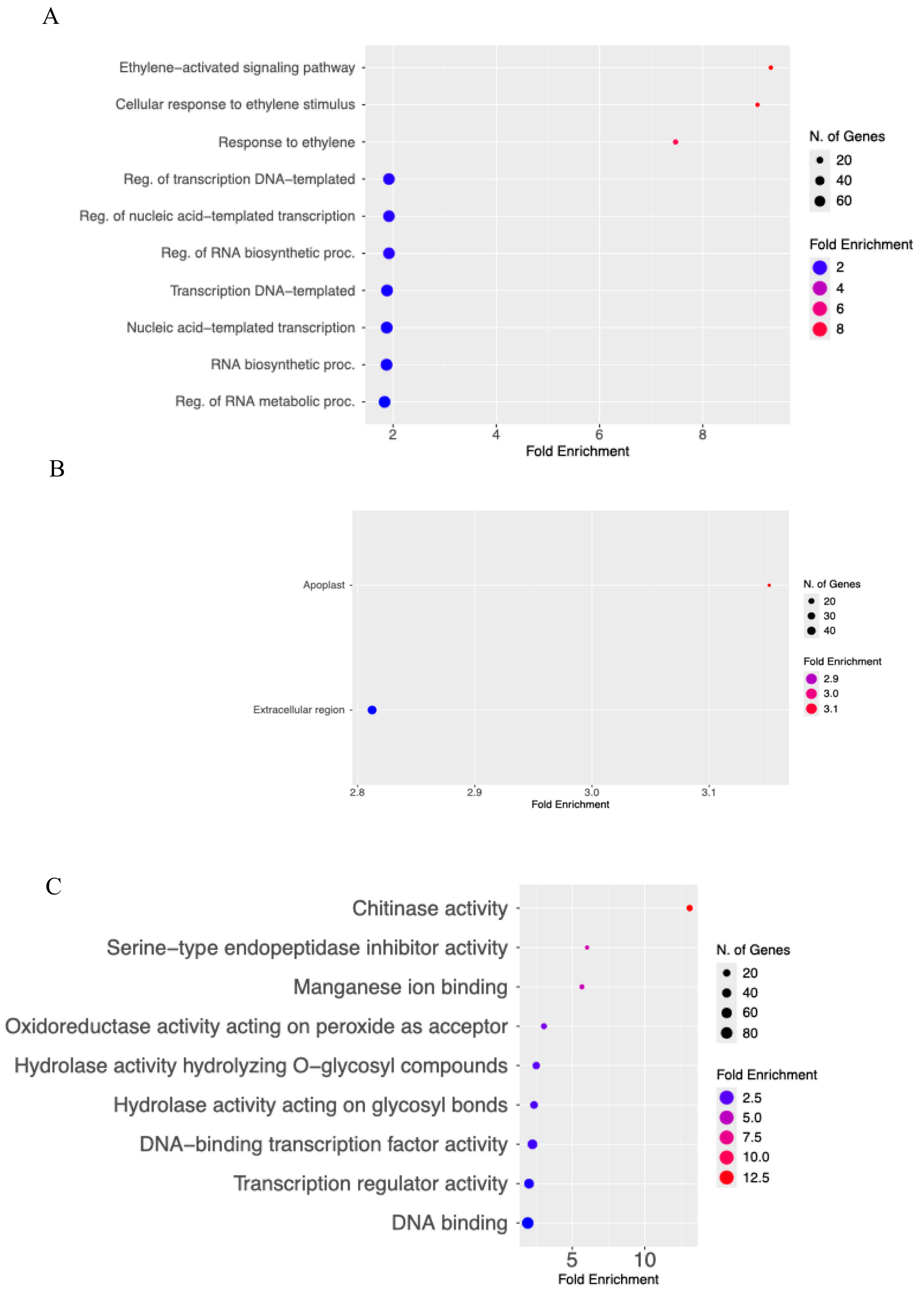

Figure S2 The GO analysis of 855 common DEGs of all tested CR-L lines. A**.** Biological process, B .Cellular component and **C.** Molecular function.

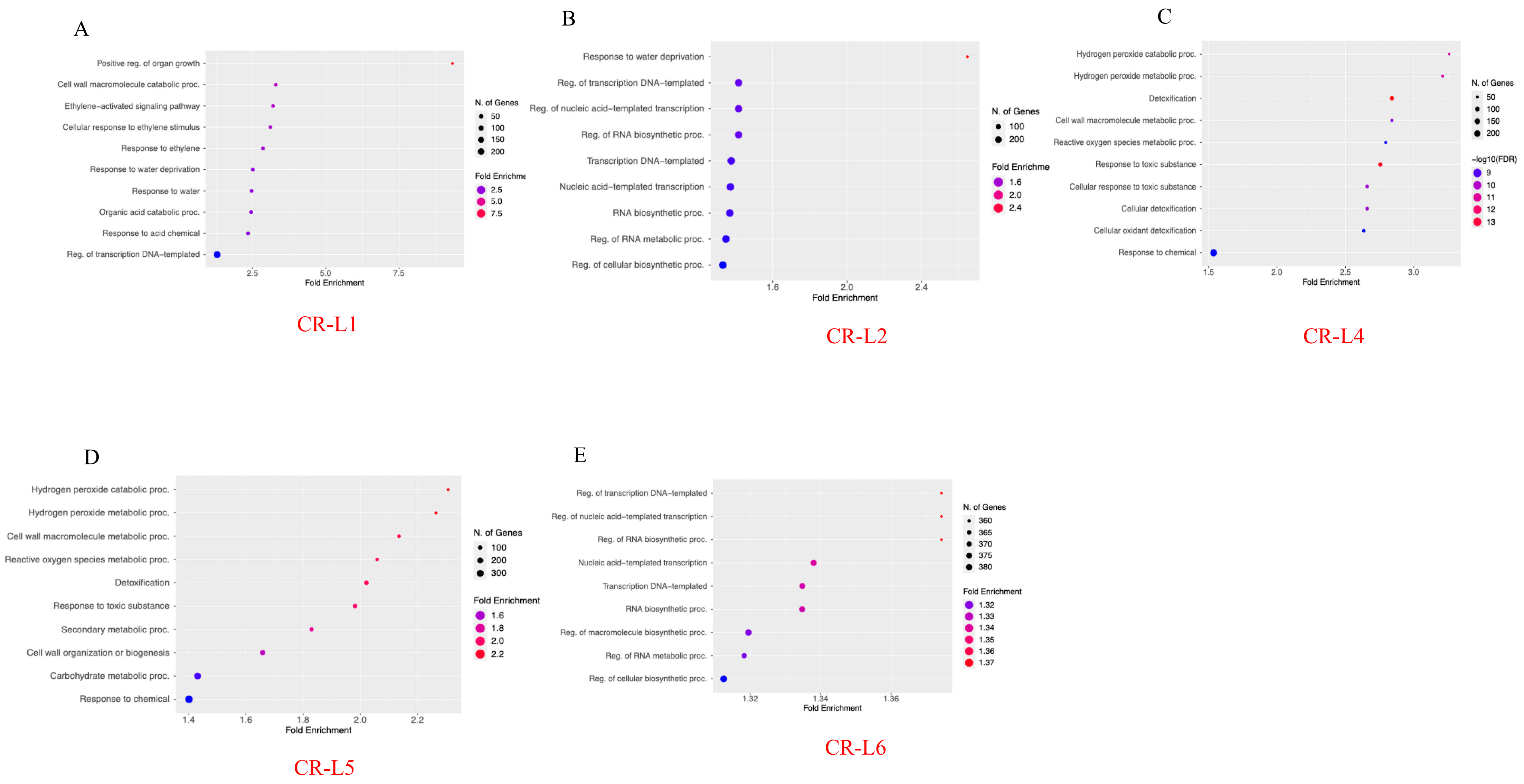

Figure S3 The GO analysis of DEGs specific for each *GS3*-editted line assigned to biological process.
A. 3926 DEGs of CR-L1, B. 4190 DEGs of CR-L2, C. 4476 DEGs of CR-L4, D. 5411 DEGs of CR-L5 and E. 6110 DEGs of CR-L6.

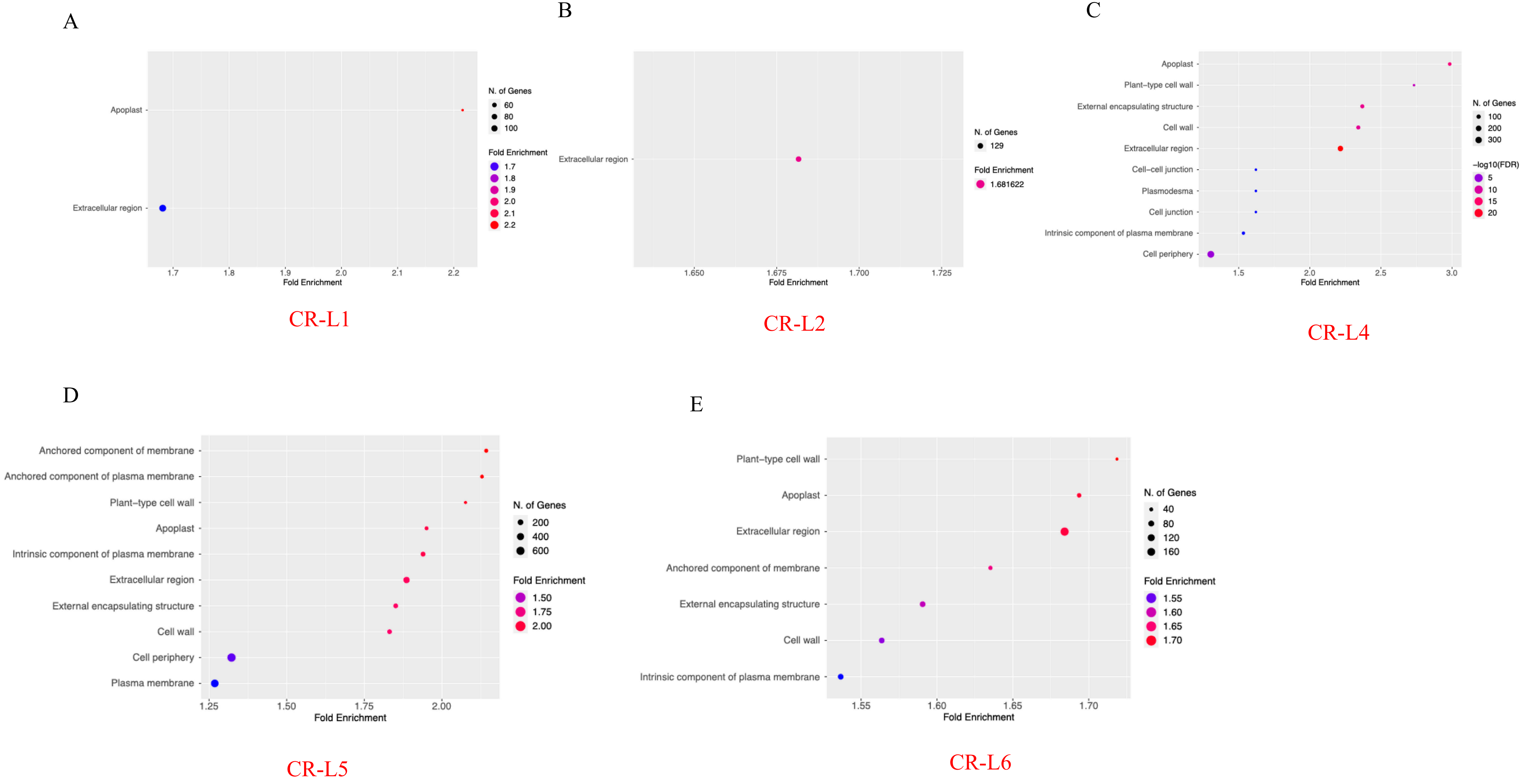

Figure S4 The GO analysis of DEGs specific for each *GS3*-editted line assigned to cellular component.
A. 3926 DEGs of CR-L1, B. 4190 DEGs of CR-L2, C. 4476 DEGs of CR-L4, D. 5411 DEGs of CR-L5 and E. 6110 DEGs of CR-L6.

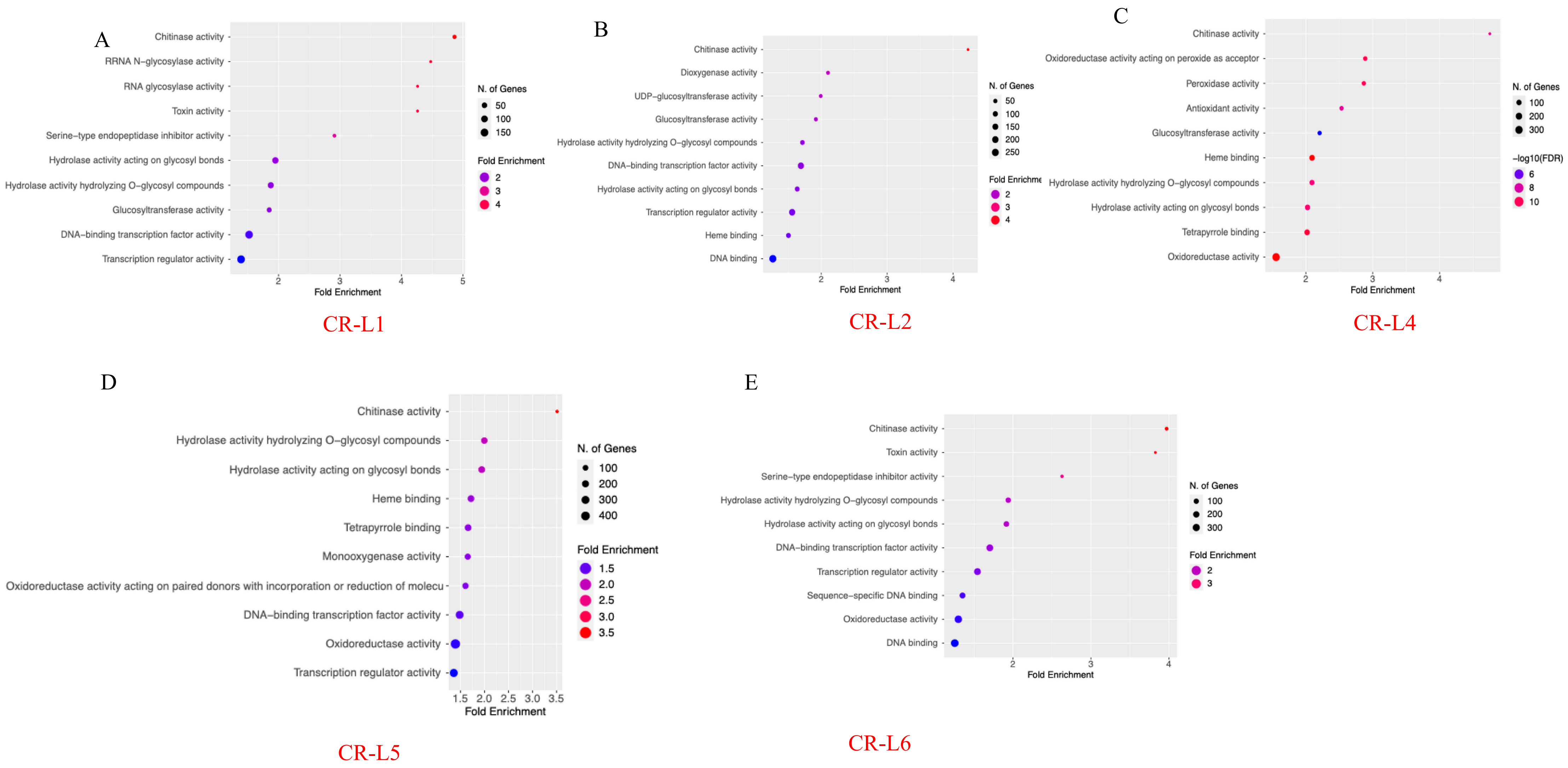

Figure S5 The GO analysis of DEGs specific for each *GS3*-editted line assigned to molecular function.
A. 3926 DEGs of CR-L1, B. 4190 DEGs of CR-L2, C. 4476 DEGs of CR-L4, D. 5411 DEGs of CR-L5 and E. 6110 DEGs of CR-L6.

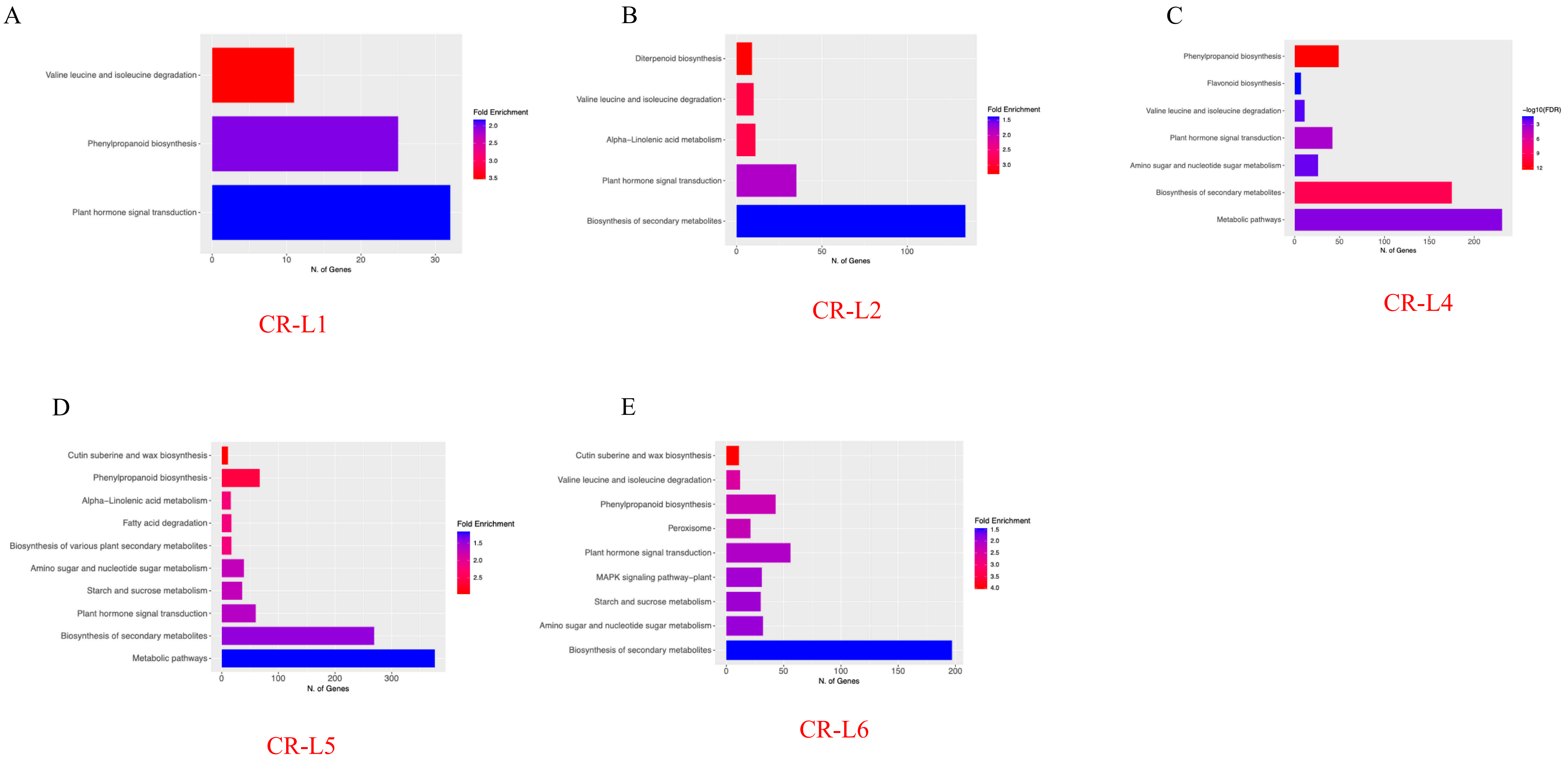

Figure S6 The KEGG analysis of DEGs specific for each *GS3*-editted lines assigned to molecular function.
A. 3926 DEGs of CR-L1, B. 4190 DEGs of CR-L2, C. 4476 DEGs of CR-L4, D. 5411 DEGs of CR-L5 and E. 6110 DEGs of CR-L6.

Table S1. The highest fold enrichment of DEGs in each CR-L line assigned to pathway using Kyoto Encyclopedia of Genes and Genomes (KEGG) pathway database.

Experiments/pathway	Gene ID	Gene	LogFC	Gene annotation
CR-L1				
osa00280 Valine leucine and isoleucine degradation	Os01g0110400	-	1.32	Similar to Acetyl-CoA C-acetyltransferase
	Os01g0269000	-	1.01	Similar to Hydroxymethylglutaryl-CoA lyase
	Os01g0314100	-	1.33	Catalytic domain of components of various dehydrogenase complexes containing protein
	Os02g0730000	ALDEHYDE DEHYDROGENASE 2A	3.23	Similar to Mitochondrial aldehyde dehydrogenase
	Os02g0817700	3-ketoacyl-CoA thiolase, 3-ketoacyl-CoA thiolase-like protein	1.20	Similar to 3-ketoacyl-CoA thiolase (Fragment)
	Os03g0231600	-	2.66	Similar to Branched-chain-amino-acid aminotransferase 3, chloroplast precursor (EC 2.6.1.42) (Atbcat-3)
	Os04g0559400	-	1.14	Similar to Branched-chain-amino-acid aminotransferase 5, chloroplast precursor (EC 2.6.1.42) (Atbcat-5)
	Os05g0125500	-	1.34	Similar to Isovaleryl-CoA dehydrogenase, mitochondrial precursor (EC 1.3.99.10) (IVD)
	Os07g0170100	-	1.26	Similar to Branched chain alpha-keto acid dehydrogenase E1 beta subunit
	Os12g0183100	-	1.93	Similar to Branched chain alpha-keto acid dehydrogenase E1-alpha subunit (Fragment)
	Os12g0605800	MCCA	1.11	Similar to 3-methylcrotonyl CoA carboxylase biotin-containing subunit (Fragment)
CR-L2				
Path:osa00904 Diterpenoid biosynthesis	Os01g0757200	GIBBERELLIN 2-OXIDASE 3	1.57	GA 2-oxidase3, GA metabolis
	Os03g0332100	P-450 714B2	1.19	Similar to Cytochrome P450 CYP714B3
	Os04g0178300	ENT-COPALYL DIPHOSPHATE SYNTHASE 4	6.56	Similar to Isoform 3 of Syn-copalyl diphosphate synthase

Experiments/pathway	Gene ID	Gene	LogFC	Gene annotation
	Os04g0179700	ENT-KAURENE SYNTHASE 4	5.98	9-beta-pimara-7,15-diene synthase, Momilactone phytoalexins biosynthesis, Defense respons
	Os05g0158600	GIBBERELLIN 2-OXIDASE 1	5.01	Similar to OsGA2ox1
	Os05g0560900	GIBBERELLIN 2-OXIDASE 8	2.26	Similar to gibberellin 2-beta-dioxygenase
	Os06g0110000	ENT-KAURENE OXIDASE	-1.25	Similar to DWARF3 (Fragment)
	Os06g0569500	ENT-KAURENE OXIDASE 4	1.15	Ent-kaurene oxidase, Diterpenoid phytoalexin biosynthesi
	Os07g0103500	GIBBERELLIN 2-OXIDASE 5	6.88	2OG-Fe(II) oxygenase domain containing protein
CR-L4 Path:osa00940 Phenylpropanoid biosynthesis	Os01g0962700	class III peroxidase 20	1.20	Similar to Peroxidase 12 precursor (EC 1.11.1.7) (Atperox P12) (PRXR6) (ATP4a)
	Os01g0963000	class III peroxidase 22	6.32	Similar to Peroxidase BP 1 precursor
	Os01g0263300	ATPASE SUBUNIT 6	-2.30	Similar to Peroxidase 72 precursor (EC 1.11.1.7) (Atperox P72) (PRXR8) (ATP6a)
	Os01g0294700	peroxidase, class III peroxidase 11	-2.61	Haem peroxidase, plant/fungal/bacterial family protein
	Os01g0327100	class III peroxidase 15	7.68	Haem peroxidase family protein
	Os01g0327400	class III peroxidase 16, Peroxidase 1	1.07	Similar to Peroxidase (Fragment)
	Os01g0283600	-	-1.61	Similar to Cinnamoyl CoA reductase
	Os02g0177600	4-COUMARATE:COENZYME A LIGASE 3	-1.04	4-coumarate:coenzyme A ligase, Lignin biosynthesis, Defense against woundin
	Os02g0236600	class III peroxidase 27	-3.40	Peroxidase P7 (EC 1.11.1.7) (TP7)
	Os02g0236800	class III peroxidase 26	-1.74	Similar to Peroxidase (EC 1.11.1.7)
	Os02g0811800	CINNAMOYL-COA REDUCTASE 10	-1.34	Similar to Cinnamoyl-CoA reductase (EC 1.2.1.44)
	Os03g0121200	class III peroxidase 33	-1.42	Similar to Peroxidase 1
	Os03g0234900	class III peroxidase 39	-1.65	Similar to Peroxidase (EC 1.11.1.7)
	Os03g0339300	class III peroxidase 41, peroxidase 8.1	-6.92	Hypothetical conserved gene
	Os03g0368000	class III peroxidase 42	4.11	Similar to Peroxidase 1
	Os03g0368300	class III peroxidase 43	3.26	Similar to Peroxidase 1
	Os03g0762300	-	3.69	Similar to Peroxidase 51 precursor (EC 1.11.1.7) (Atperox P51) (ATP37)

Experiments/pathway	Gene ID	Gene	LogFC	Gene annotation
	Os03g0762400	class III peroxidase 51	1.71	Similar to Peroxidase2 precursor (EC 1.11.1.7)
	Os04g0518400	Phenylalanine ammonia-lyase 07	-3.48	Similar to Phenylalanine ammonia-lyase (Fragment)
	Os04g0688600	class III peroxidase 63	-7.04	Peroxidase (EC 1.11.1.7)
	Os04g0688200	class III peroxidase 60, peroxidase 1	-5.24	Similar to Peroxidase (EC 1.11.1.7)
	Os04g0688300	class III peroxidase 61, Peroxidase 2	1.59	Haem peroxidase, plant/fungal/bacterial family protein
	Os04g0689000	class III peroxidase 64	2.02	Similar to Peroxidase (EC 1.11.1.7)
	Os05g0135500	class III peroxidase 71, Peroxidase 63	2.97	Haem peroxidase family protein
	Os05g0162000	class III peroxidase 72	2.52	Similar to Peroxidase (Fragment)
	Os05g0363100	-	3.38	Similar to Monoglyceride lipase
	Os05g0427400	Phenylalanine ammonia lyase	3.22	Similar to Phenylalanine ammonia-lyase
	Os05g0494000	p-coumaroyl shikimate/quinate 3-hydroxylase 1	-1.10	p-Coumaroyl ester 3-hydroxylase, Determination of lignin content, composition and the degree of cell wall cross-linkin
	Os05g0499300	PEROXIDASE 1	-4.01	Similar to Peroxidase (EC 1.11.1.7)
	Os05g0499400	class III peroxidase 75	-2.98	Haem peroxidase family protein
	Os06g0274800	class III peroxidase 77	-8.06	Similar to Peroxidase 11 precursor (EC 1.11.1.7) (Atperox P11) (ATP23a/ATP23b)
	Os06g0546500	class III peroxidase 88	-1.58	Similar to Class III peroxidase GvPx2b (Fragment)
	Os06g0681600	class III peroxidase 89	-1.08	Haem peroxidase family protein
	Os06g0695400	class III peroxidase 91	7.18	Haem peroxidase family protein
	Os07g0115300	class III peroxidase 98	-3.22	Similar to Peroxidase2 precursor (EC 1.11.1.7)
	Os07g0638300	-	5.02	Similar to 1-Cys peroxiredoxin
	Os07g0676900	class III peroxidase 109, peroxidase 2	-5.47	Similar to Peroxidase (EC 1.11.1.7)
	Os07g0677300	PEROXIDASE GX9	-6.50	Peroxidase
	Os07g0677600	class III peroxidase 115	-6.37	Similar to Cationic peroxidase
	Os08g0157500	O-METHYLTRANSFERASE 9	-2.22	Caffeic acid O-methyltransferase, Flavonoid 3'-O-methyltransferase, Melatonin biosynthesis, Tricin biosynthesi
	Os08g0245200	4-COUMARATE:COENZYME A LIGASE 1	-6.50	4-coumarate:coenzyme A ligase, Lignin biosynthesi
	Os08g0302000	class III peroxidase 118	-2.97	Similar to Peroxidase 40 precursor (EC 1.11.1.7) (Atperox P40)

Experiments/pathway	Gene ID	Gene	LogFC	Gene annotation
	Os09g0127300	-	-6.21	NAD(P)-binding domain containing protein
	Os10g0109300	class III peroxidase 125	-1.54	Similar to Peroxidase (EC 1.11.1.7)
	Os10g0109600	PEROXIDASE A	5.81	Peroxidase (EC 1.11.1.7)
	Os11g0112200	class III peroxidase 131	-5.64	Similar to Cationic peroxidase 1 precursor (EC 1.11.1.7) (PNPC1)
	Os11g0210100	class III peroxidase 132, class III peroxidase 133	-2.03	Similar to Class III peroxidase 133
	Os11g0661600	class III peroxidase 134	-4.78	Similar to Peroxidase (EC 1.11.1.7)
	Os12g0530100	class III peroxidase 138	-4.73	Similar to Peroxidase 24 precursor (EC 1.11.1.7) (Atperox P24) (ATP47)
CR-L5 Path:osa00073 Cutin suberine and wax biosynthesis	Os01g0924933	DEFECTIVE POLLEN WALL 2	-2.12	Transferase family protein
	Os02g0666500	-	-1.25	Similar to Cytochrome P450
	Os03g0168600	CYTOCHROME P450 HYDROXYLASE 704B2	-2.01	Cytochrome P450 protein, Anther cutin biosynthesis and pollen exine formatio
	Os04g0353600	RAPID ALKALIZATION FACTOR 2	6.30	Similar to OSIGBa0092G14.1 protein
	Os04g0354600	ECERI1FERUM4, ECERI1FERUM 4	-1.18	Similar to Acyl CoA reductase-like protein
	Os04g0511200	EF-hand, abscisic acid responsive 27-kDa protein	-1.33	EFA27 for EF hand, abscisic acid, 27kD
	Os04g0560100	P-450 86A7-1	-1.63	Cytochrome P450 family protein
	Os06g0254600	-	-2.67	Caleosin related family protein
	Os08g0401500	-	-2.80	Similar to HOTHEAD protein precursor (ADHESION OF CALYX EDGES protein)
	Os09g0363900	ONION3	-1.05	Putative omega-alcohol dehydrogenase, Biosynthesis of long-chain fatty acids, Organ separation, Shoot developmen
	Os10g0486100	-	-4.53	Cytochrome P450-like protein (CYP86B1)
CR-L6 Path:osa00073 Cutin suberine and wax biosynthesis	Os01g0924933	DEFECTIVE POLLEN WALL 2	-4.12	Transferase family protein
	Os02g0666500	-	-1.06	Similar to Cytochrome P450

Experiments/pathway	Gene ID	Gene	LogFC	Gene annotation
	Os03g0167600	MALE STERILITY 2, male sterility protein 2, defective pollen wall	1.80	Similar to Male sterility protein 2
	Os03g0168600	CYTOCHROME P450 HYDROXYLASE 704B2	-3.35	Cytochrome P450 protein, Anther cutin biosynthesis and pollen exine formatio
	Os04g0353600	RAPID ALKALIZATION FACTOR 2	3.12	Similar to OSIGBa0092G14.1 protein
	Os04g0354600	ECERI1FERUM4, ECERI1FERUM 4	-1.19	Similar to Acyl CoA reductase-like protein
	Os04g0511200	EF-hand, abscisic acid responsive 27-kDa protein	10.51	EFA27 for EF hand, abscisic acid, 27kD
	Os04g0560100	P-450 86A7-1	-1.02	Cytochrome P450 family protein
	Os08g0401500	-	-1.83	Similar to HOTHEAD protein precursor (ADHESION OF CALYX EDGES protein)
	Os09g0363900	ONION3	-1.21	Putative omega-alcohol dehydrogenase, Biosynthesis of long-chain fatty acids, Organ separation, Shoot developmen
	Os10g0486100	-	-3.93	Cytochrome P450-like protein (CYP86B1)

Yellow represented common DEGs in all CR-L lines, and blue represented specific DEGs for each CR-L lines.
